# Developing and Evaluating a Target-Background Similarity Metric for Camouflage Detection

**DOI:** 10.1371/journal.pone.0087310

**Published:** 2014-02-03

**Authors:** Chiuhsiang Joe Lin, Chi-Chan Chang, Bor-Shong Liu

**Affiliations:** 1 Department of Industrial Management, National Taiwan University of Science and Technology, Taiwan, Republic of China; 2 Department of Industrial Engineering and Management, St. John’s University, New Taipei City, Taiwan, Republic of China; University of Sussex, United Kingdom

## Abstract

**Background:**

Measurement of camouflage performance is of fundamental importance for military stealth applications. The goal of camouflage assessment algorithms is to automatically assess the effect of camouflage in agreement with human detection responses. In a previous study, we found that the Universal Image Quality Index (UIQI) correlated well with the psychophysical measures, and it could be a potentially camouflage assessment tool.

**Methodology:**

In this study, we want to quantify the camouflage similarity index and psychophysical results. We compare several image quality indexes for computational evaluation of camouflage effectiveness, and present the results of an extensive human visual experiment conducted to evaluate the performance of several camouflage assessment algorithms and analyze the strengths and weaknesses of these algorithms.

**Significance:**

The experimental data demonstrates the effectiveness of the approach, and the correlation coefficient result of the UIQI was higher than those of other methods. This approach was highly correlated with the human target-searching results. It also showed that this method is an objective and effective camouflage performance evaluation method because it considers the human visual system and image structure, which makes it consistent with the subjective evaluation results.

## Introduction

To defend against an enemy’s high-tech weaponry for reconnaissance and attack, and to protect an army’s strategic and tactical objectives, it is essential to use a large range of camouflage patterns. In modern high-tech warfare, it is important to use camouflage to hide high-priced weapons and equipment [1]. Therefore, in order to effectively exert a camouflage effect, the camouflage should be developed through proper design methods. However, it is still difficult to accurately evaluate the effectiveness of camouflage. The assessment parameters and detection model must be systematic and rational. Traditionally, camouflage assessment has used military observers in specific settings to assess the performance of camouflage [2]–[3]. However, this approach is expensive, time consuming, and complicated, and the experimental conditions are difficult to control. In order to provide a new method to improve the objectivity and validity of camouflage evaluations, the research and technology organization (RTO) of NATO presented photo-simulation [4]. In photo-simulation, a set of image slides of camouflaged targets is presented in highly similar circumstances in the laboratory and in the field for observers to identify the performance of the designs visually and subjectively [5]–[7].

Photo-simulation, which uses various types of digital image processing to simulate the objective world, can work directly or indirectly on the perception of the human eye. Image quality assessment has traditionally attempted to quantify the visibility of differences between a distorted image and a reference image. In past studies, many researchers have contributed significantly to the development of image-quality assessment algorithms, and these methods have been used in developing a camouflage assessment method [8]–[10]. The goal is to develop an image assessment algorithm consistent with subjective human visual judgment that has objectively quantified characteristics. An image-quality assessment algorithm in close agreement with the human vision system (HVS) could also be made publicly available for camouflage assessment.

The image quality assessment algorithm plays an important role in many digital image processing applications, and it can be used in video image quality evaluation, monitor image quality assessment, or digital image quality appraisal. Image quality assessment can be divided into two types: subjective evaluation and objective assessment [11]–[12]. Subjective evaluation uses multiple observers to judge the quality of the test images and subjectively score a comprehensive evaluation on the weighted average [13]. But subjective evaluation takes much time, is high in cost, and is greatly restricted in its practical applications. Objective assessment, on the other hand, uses numerical calculation methods to quantify the image quality indicators [14]. In recent years, several objective assessments for image quality analysis have been proposed, such as the Mean Square Error (MSE) and Peak Signal Noise Ratio (PSNR) [15]. MSE and PSNR have recently been employed as the standard metrics for algorithm design, parameter optimization, and system testing in most image quality assessments. Although PSNR and MSE are simple to calculate, they do not match human psychophysical performance well [16]–[17]. To rectify this metrics based on error sensitivity have been comprehensively developed by considering more characteristics of the HVS, such as the just noticeable difference (JND) threshold and normalization [18]. A fundamentally different framework, called the Universal Image Quality Index (UIQI), is based on the assumptions of the HVS [16]; [19], and it is highly adapted for extracting statistical structural information. In our previous study [20], we investigated the effectiveness of different camouflage designs using this index. Camouflaged human targets were presented on a natural landscape and the targets were designed to be similar to the landscape background with different levels of background similarity as estimated by the image index. The target was presented in front of the observer (central 0°) or at different angles in the left (−7°, −14°, −21°) or right (+7°, +14°, +21°) visual fields. The observer had to detect the target using peripheral vision if the target appeared in the left and right visual fields. The camouflage effectiveness was assessed by detection hit rates, detection times, and subjective ratings on detection confidence and task difficulty. The study showed that the psychophysical measures correlated well with the image similarity index, suggesting potential as an efficient camouflage effectiveness assessment tool if the relationship between the psychophysical results and the index can be quantified. In another study [21], several different camouflage design patterns against two different backgrounds were compared using human performance-based measures (hit rate and detection time) and eye movement-based measures. This study found that eye movement data significantly enhanced the sensitivity of the comparisons between the different designs in the evaluation of camouflage performance. In particular, two eye movement measures, first saccade amplitude to the area of interest and fixation duration in the area of interest, were suggested for the finer comparison of camouflage designs. By analyzing the eye movement data, the study further argued that detectability seemed to play a more important role than discriminability in camouflaged target search. It suggested that in camouflage design, the first goal should be to decrease detectability which is mostly associated with contrast design (Cai et al., 2012; Lindqvist et al., 2007). The second goal was then to prevent discrimination which was related to the structural differences between the target and the background. Thus, contrast and structural differences could be considered as the major components in determining the target-background similarity which may be the key to the success of camouflage design. In this paper, we present a computation method that arouse from the above ideas to evaluate camouflage performance adapting from existing image fusion evaluation metrics, wherein psychophysical data and eye movement measures as suggested in [21] were included as the guidance and support for comparative assessment of these metrics. Using similar approach as in [21], the experiment was performed in the overall context of search and detection of camouflaged targets in a natural scene, where human participants were required to look for and detect camouflaged human-shaped military targets. This work attempts to find an index, and evaluate index for effectively and easily calculating the performance of camouflage in terms of human perception. The following experimental results performed using fovea vision show that UIQI metrics perform well for camouflage target searching assessment and are closer to human detection performances compared to other available indexes.

## Method

The following gives brief introduction to several indexes available in image quality classification literature and how they are adapted in the camouflage similarity context.

### MSE

MSE is mean-squared error measures. MSE is defined as [22].
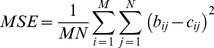
(1)


Where b_ij_ is the Red, Green, Blue (RGB) grey level of (i,j) point of the background image; c_ij_ is the Red, Green, Blue (RGB) grey level of an (i,j) point of the camouflage image; b_ij_ and c_ij_ represent RGB grey scale values from 0 to 255; and M×N is the pixels’ number in the image [13 pixels × 50 pixels]. It shows that a lower MSE value provides a higher image similarity.

### PSNR

PSNR is peak signal to noise ratio measures. PSNR is defined as the ratio of peak signal power to average noise power [22].
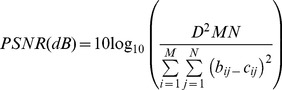
(2)


Where D is the maximum peak-to-peak swing of the pixel value (255 for 8-bit images), and b_ij_ is the Red, Green, Blue (RGB) grey level of (i,j) point of the background image; c_ij_ is the Red, Green, Blue (RGB) grey level of an (i,j) point of the camouflage image; b_ij_ and c_ij_ represent RGB grey scale values from 0 to 255; and M×N is the pixels’ number of the minimum rectangle which contains the camouflage image [13 pixels × 50 pixels in this study]. It shows that a higher PSNR value provides a higher image similarity.

### UIQI

The main function of the human visual system is to extract the structural information of an image, and use this information to form the best approximation of the image perceived quality. Based on this principle, Wang and Bovik proposed a universal image quality index (UIQI) for classification of images of different distortion level [16]. This index UIQI was adapted in this paper to classify and calculate the similarity between the background (b) and the camouflage designs (c).

The UIQI is defined as
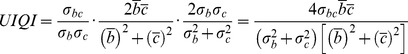
(3)where



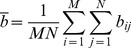
(4)

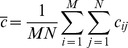
 (5)
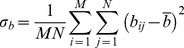
(6)

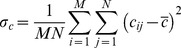
(7)

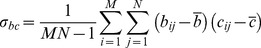
(8)where b_ij_ is the Red, Green, Blue (RGB) grey level of (i,j) point of the background image; c_ij_ is the Red, Green, Blue (RGB) grey level of an (i,j) point of the camouflage image; b_ij_ and c_ij_ represent RGB grey scale values from 0 to 255; and M×N is the pixels’ number in the image [13 pixels × 50 pixels]. The UIQI is calculated for the same area in both the target and the background. In Eq (3), the first component is the correlation coefficient between the camouflage and background images, and the second component is to calculate the similar degree of the mean luminance between the camouflage and background images. The third component calculates how close the contrast is between the camouflage and background. This is an improvement over the traditional method which only considers luminance. The UIQI considers correlation coefficient, luminance, and contract information, which would make the UIQI potentially more similar to HVS processing, although it should be noted that this treatment still cannot be said to explicitly base on the HVS processing. The dynamic range of UIQI is [−1, 1]. The best value is 1 and is achieved if and only if background image and camouflage are the same.

### Experiments

We conducted a series of experiments to check the validity of the proposed UIQI metrics, according to the NATO seminar SCI-012 camouflage search evaluation criteria [4]; [23]. An eye tracker was used record eye movement information in the experiment. The purpose of this experiment was to test whether the proposed algorithm included subjective evaluation and whether the proposed objective metric was an effective camouflage performance predictor.

### Image Stimuli

**Figure 1:**
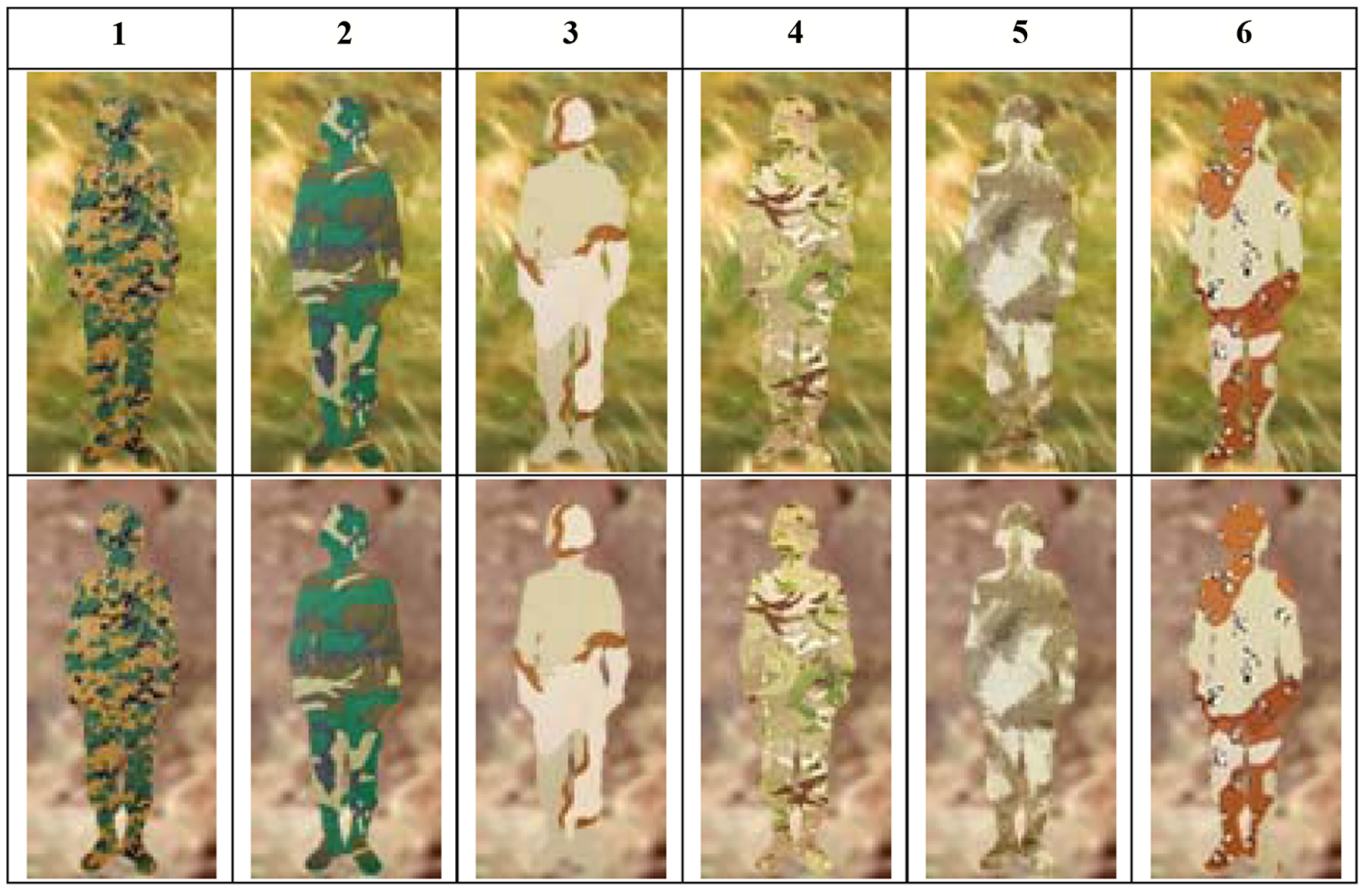
Camouflage types shown with the two backgrounds (top is grassy, bottom is rocky). (1) MARPAT, (2) ERDL patterns, (3) Three-color Desert, (4)Muti-cam, (5)A-TACS, (6) Six.-color Desert.

**Figure 2:**
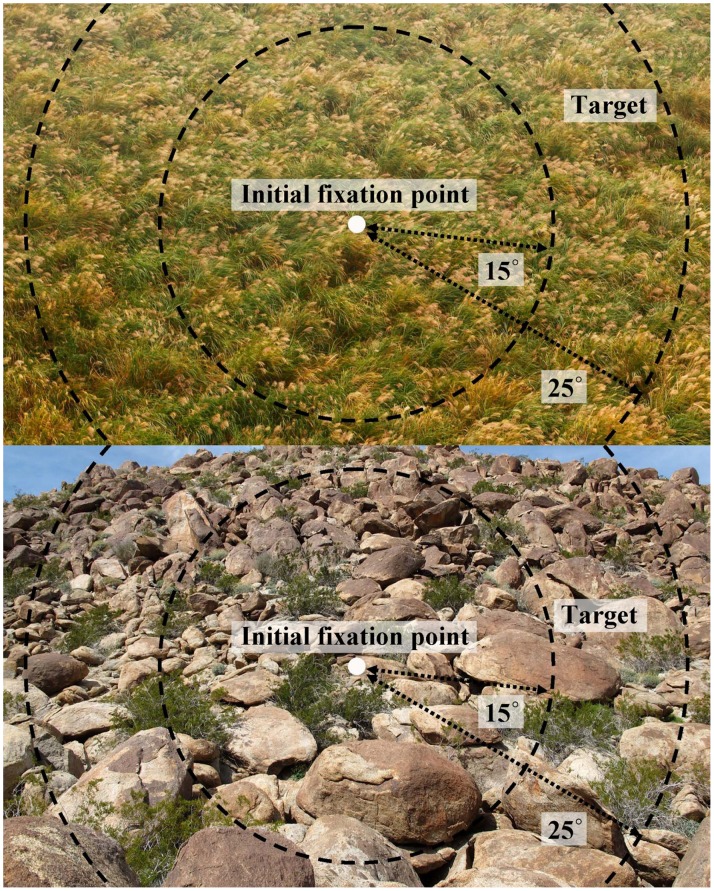
Background of grassy (top) and rocky (down) environment.

In this study, we applied the UIQI method with MATLAB to calculate the similarity between camouflage and background. Both the camouflage and background images were the same size, and there was a natural one-to-one correspondence between pixels in the same position. We also used MSE and PSNR as examples to calculate the camouflage similarity metrics of 6 different types of camouflage in four different positions on grassy and rocky backgrounds, and from the performance comparison demonstrated the proposed usage and its effectiveness. All the algorithms are either publicly available on the Internet or available from the authors, and all of the images were tested using the code available on the author’s website [12]. In this study, we also used Adobe Photoshop to design experimental stimuli. Six camouflage patterns are shown in Figure 1. These camouflage patterns mirrored designs with micro-patterns that were synthesized using different techniques [24]–[26]. Background images were collected in grassy and rocky environments of interest in Taiwan, as shown in Figure 2. These two backgrounds are common training fields. All the stimuli and background collection process and definition are following the guideline- Photosimulation camouflage detection test from U.S. Army Natick Soldier Research, Development and Engineering Center [25]–[26]. All the camouflage and background image was shoot by Canon EOS 5D Mark II, and we use ICC workflows to calibrate the image from digital single-lens reflex camera and display screen. This method could serve several color devices to a standard color space. The image simulated target detection at a distance of 75 meters, and all the stimulus images were resized to 1920 pixels (518.4 mm) by 1200 pixels (324 mm) for use in the experiment.

### Participants

A total of 15 male students from the National University of Defense Technology, aged between 19 and 23 years old, participated in the study. All participants had more than one year of military training and education. All participants had (corrected to) normal vision and none of the participants had a history of color-blindness or vision-related illness. The experiment had been conducted according to the ethical guidelines laid down by the Research Ethics Committee of National Taiwan University. The Research Ethics Committee of National Taiwan University approved this study and the consent procedure. All participants understood the purpose of the experiment, and participants gave their written informed consent to participate in this study.

### Apparatus

The EyeLink II Eyetracker (SR Research, Osgoode, Ontario, Canada) was used to monitor and record the participants’ eye movements. It was linked to a display monitor in order to provide gazing positions and saccadic data in real time. A host PC was used to respond to the displayed stimulus and to trigger the Eyetracker in each trial. Stimuli were shown on an industrial color correction monitor (EIZO color edge 243 W; Display area: 518.4 mm× 324 mm). The participant’s chair and monitor stand were adjusted such that the center of the display monitor was at the participant’s eye level at a distance of 55 cm from the participant’s eyes.

### Measures

The six stimulus camouflage patterns were tested by the participants in the searching task, and their responses were recorded. In this study, all the experimental results were used to compare camouflage assessment metrics. The dependent variables were hit rate, detection time, difficulty, and first saccade amplitude to interest area. The interest area is defined to be the minimum rectangle which contains the camouflage image, as shown in figure 2. “Hit rate” was defined as the percentage of times that the camouflaged targets were correctly hit by the mouse, and detection time was the duration from the beginning to hit or quit by observers. Task difficulty ratings on a seven-point rating scale were collected at the end of each trial. For the ratings of difficulty of target detection, “1” represented the easiest workload and “7” the most difficult workload. Past research has shown that the ideal searching trajectory is that the pupil jumps directly toward the target in a very short time, and a large saccade amplitude to target also indicates that the user has high skill or that the target provides effective clues [27]–[28]. A longer fixation duration indicates that the information is more difficult to recognize or understand [27], [29]. In this paper, we used the first saccade amplitude to interest area to assess camouflage performance in peripheral vision [20] and fixation duration in interest area (IA) to assess camouflage performance in fovea vision [30]–[32].

### Procedures

**Figure 3:**
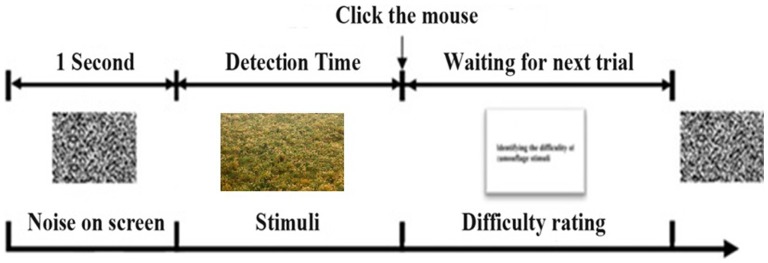
Data collecting procedure and the definition of detection time.

Before the experiment, all participants were introduced to the procedures, and they were presented with a sample image to familiarize them with the visual search procedure. Eye fixation was calibrated manually before the experiment, and only calibration values with a mean error <0.5° were accepted. This calibration accuracy was checked at the beginning of each trial to check the eye-tracker’s correction for drifts and slips. The order of presentation was randomized to avoid sequence effects. Participants were told that every task had a target, and that when they found the camouflaged human shape, they should click it with the mouse to confirm it as quickly and as accurately as possible. When the participants clicked the mouse button, the display screen would show a question asking the participants to rate the level of difficulty of the task. The experiment had no time limit; if a participant could not find the target, he could press a space key to quit the search. Participants had a five-minute rest period in the middle of the experiment to reduce the effects of possible visual fatigue. Figure 3 shows the procedure of the experiment.

## Results

**Figure 4:**
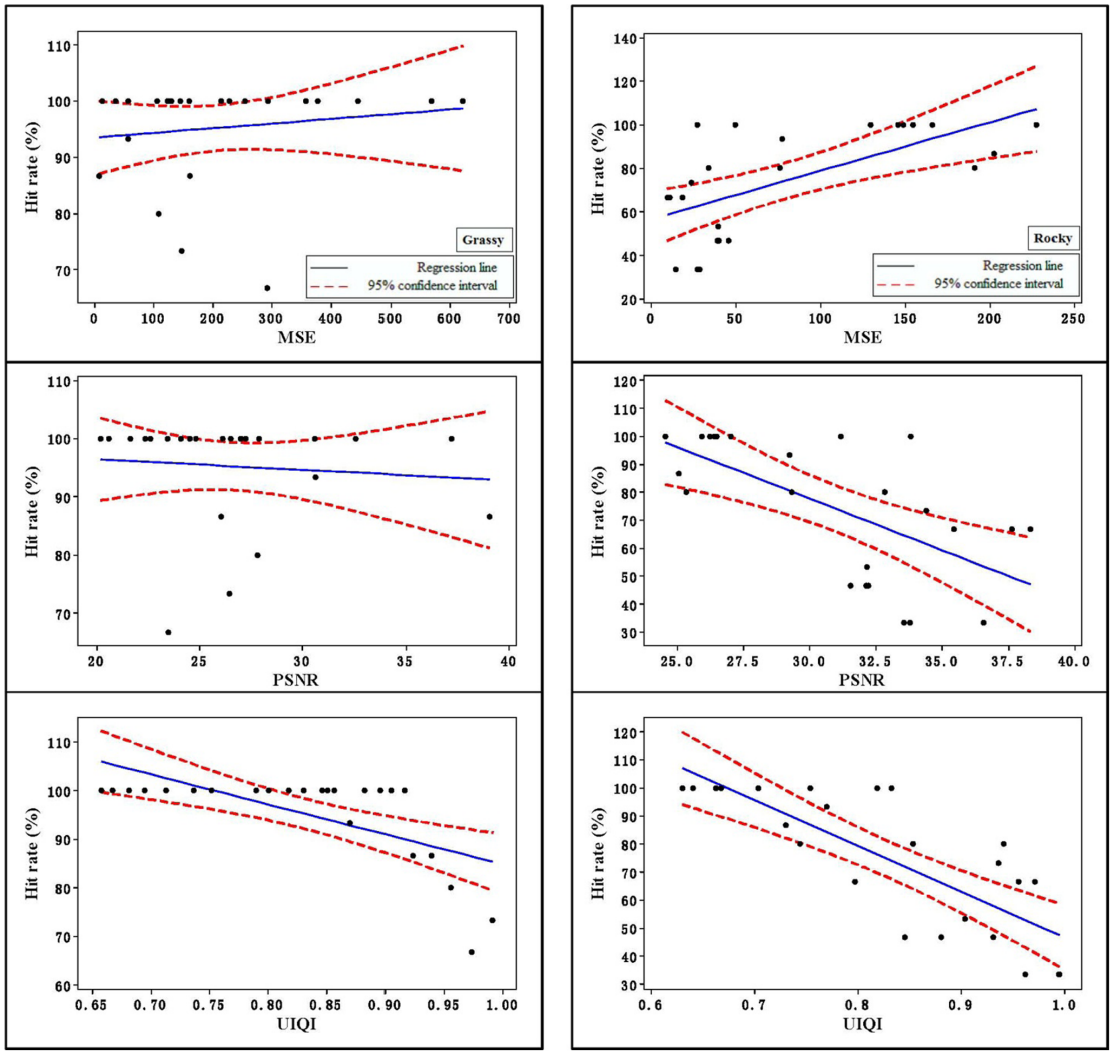
The relationships of MSE, PSNR, and UIQI vs. hit rate.

**Figure 5:**
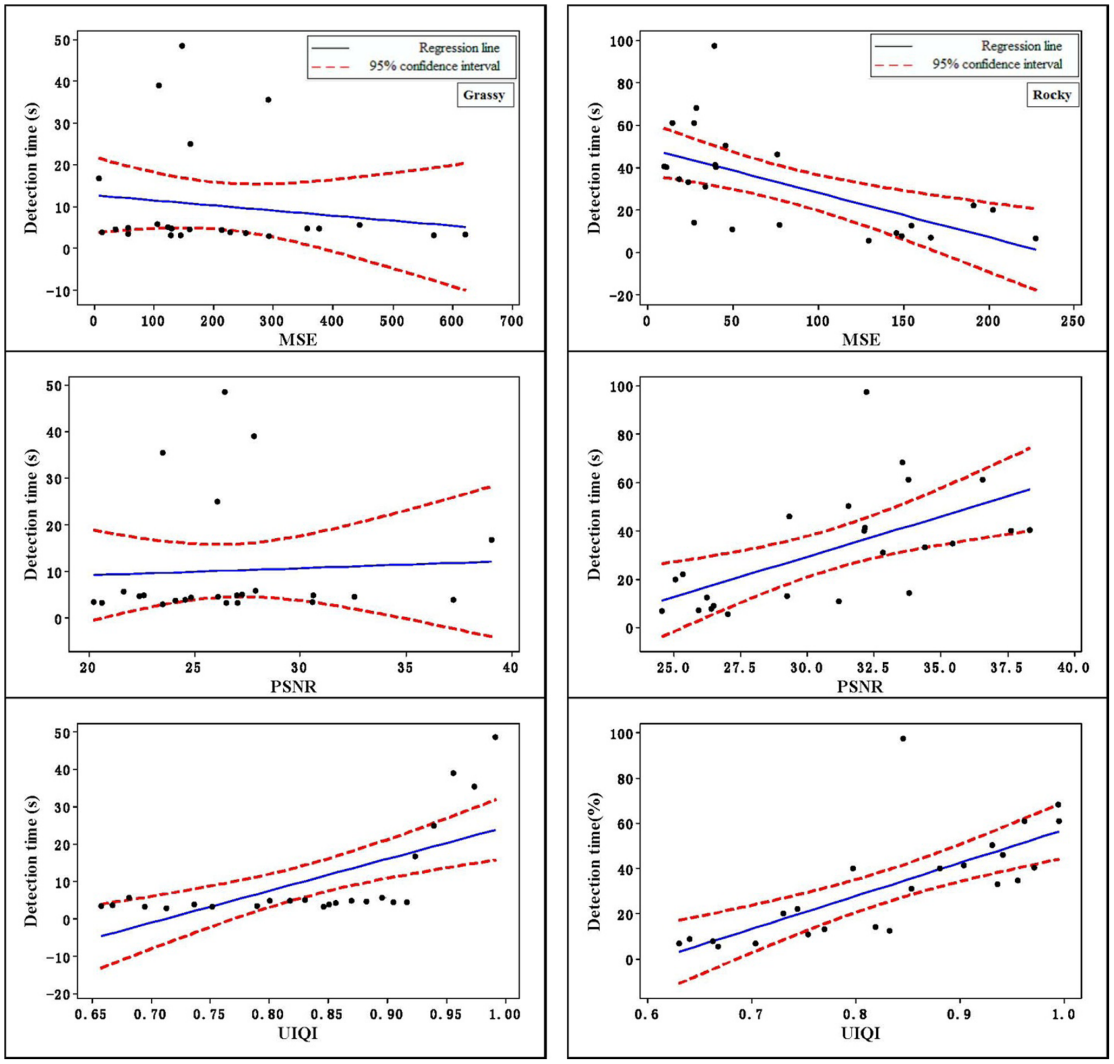
The relationships of MSE, PSNR, and UIQI vs. detection time.

The data were analyzed with Minitab, and all the image-quality assessment variables available from the literature (MSE, PSNR, and UIQI) were used individually as predictors for camouflage performance. Five camouflage performance measures (hit rate, detection time, difficulty rating, first saccade to interest area, and fixations in IA) were considered. The Pearson correlation results between each of the predictors and the performance measure are shown in Table 1. The Pearson correlation coefficients is an indicator of how well the predictor could be applied to predict the performance measure; i.e., the higher the Pearson correlation coefficients, the better the predictor in predicting performance. Table 1 shows that on the grassy and rocky background, the correlation coefficients of UIQI was higher than those of the other two predictors to predict hit rates, and Figure 4 also shows that UIQI had a more linear trend than the others. Similarly, UIQI outperformed the other predictors in predicting detection time. Whether the Pearson correlation had statistical significance was tested, and the results are shown in Table 1 and Figure 5. It can be seen from the table that most of the Pearson correlation passed the significance test; that is, the predictor explained a significant degree of the camouflage task performance.

**Table 1:** Summary results of correlations for each of the four dependent variables as a function of each of the three predictors.

Source of variance	Hit rate, %	Detection time, msec.	Difficulty	First saccade AMP to IA	Fixation duration in IA
MSE(G)	0.146	0.155	0.240	0.319	0.259
PSNR(G)	0.091	0.056	0.128	0.212	0.265
UIQI(G)	0.654**	0.659**	0.775**	0.762**	0.653**
MSE(R)	0.633**	0.616**	0.730**	0.737**	0.317
PSNR(R)	0.634**	0.591**	0.689**	0.725**	0.290
UIQI(R)	0.791**	0.730**	0.902**	0.890**	0.444*

Significance of the Pearson correlation: *p<.05, **p<.01.

(G) Grass background, (R) Rocky background.

**Figure 6:**
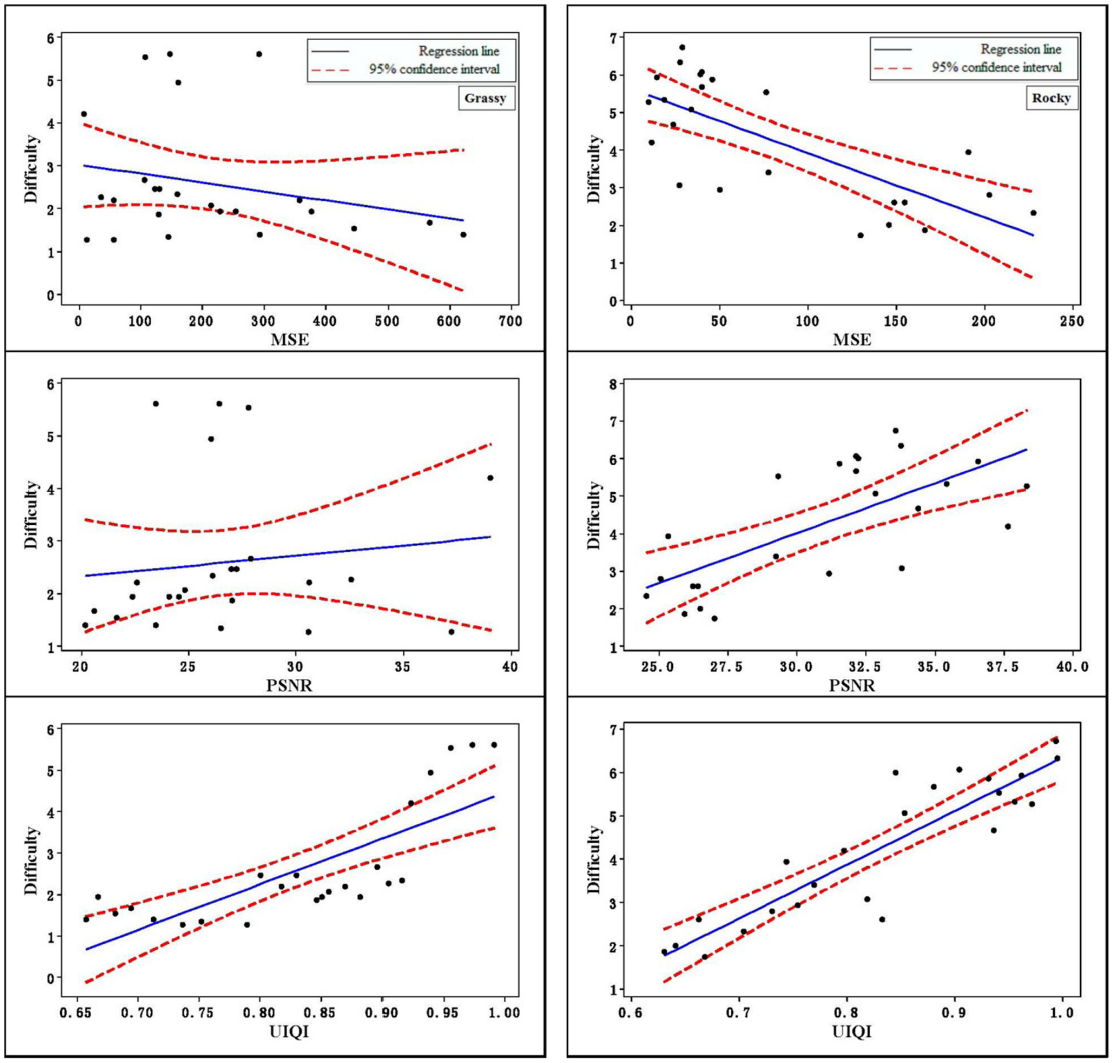
The relationships of MSE, PSNR, and UIQI vs. difficulty.

The relationships between each of the predictors and difficulty are shown in Table 1 and Fig. 6. Table 1 showed that UIQI had the highest Pearson correlation coefficients to predict detection time and to explain the relationship between the difficulty and the UIQI. From Figure 6, it is also clear that UIQI had a more linear tendency.

**Figure 7:**
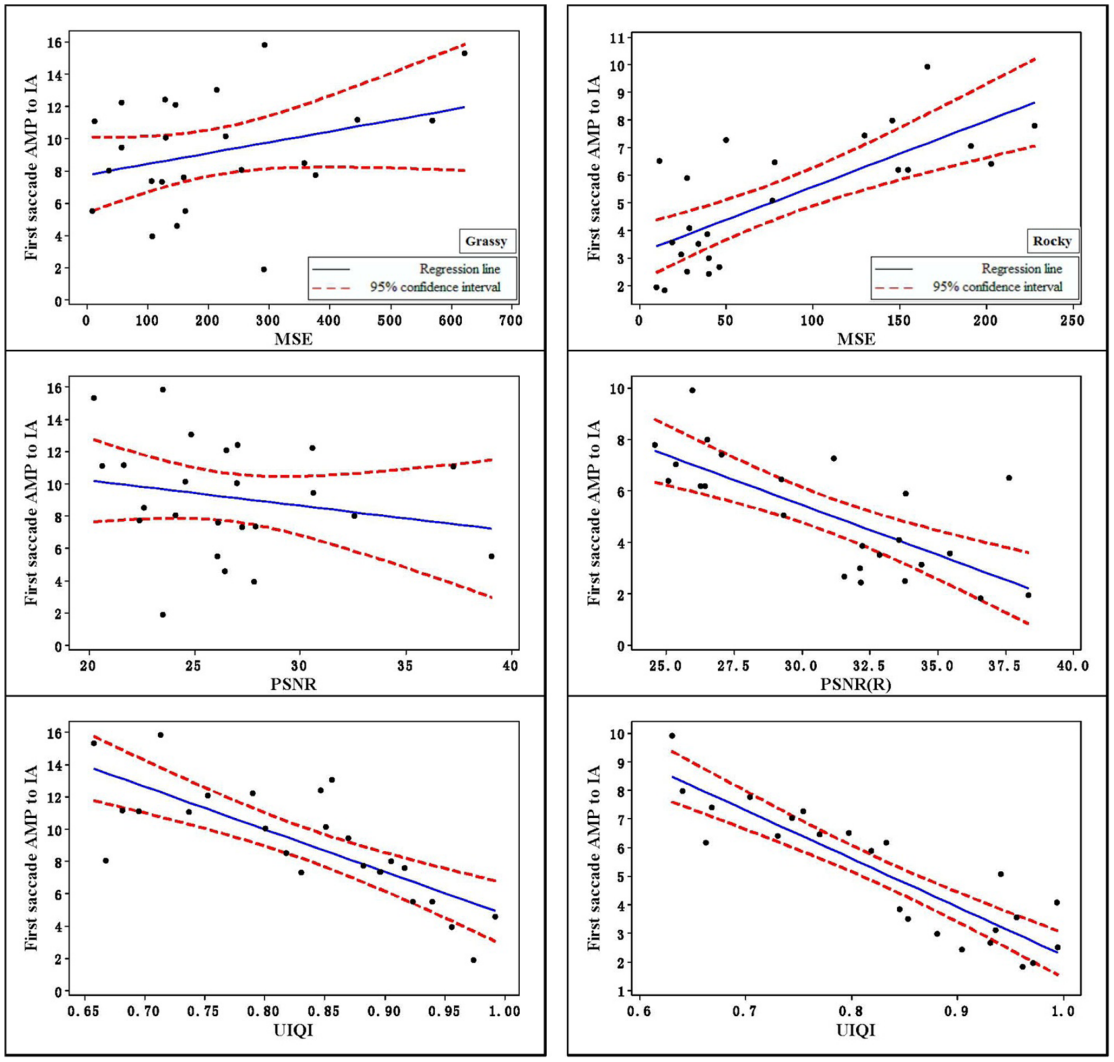
The relationships of MSE, PSNR, and UIQI vs. first saccade AMP to IA.

When there was a fixation within the interest area, the saccade amplitude into the interest area was collected. The mean first saccade amplitude is shown in Figure 7. The Pearson correlation coefficients yielded a significant relationship between the three factors of computer vision and first saccade amplitude. From the results in the table and figures, we can see that MSE and PSNR had the greatest deviation from the results from human subjects because only local information of an image was considered. UIQI includes image structure information in the image fusion quality metrics, and UIQI was more straightforward than the implementation of the others.

**Figure 8:**
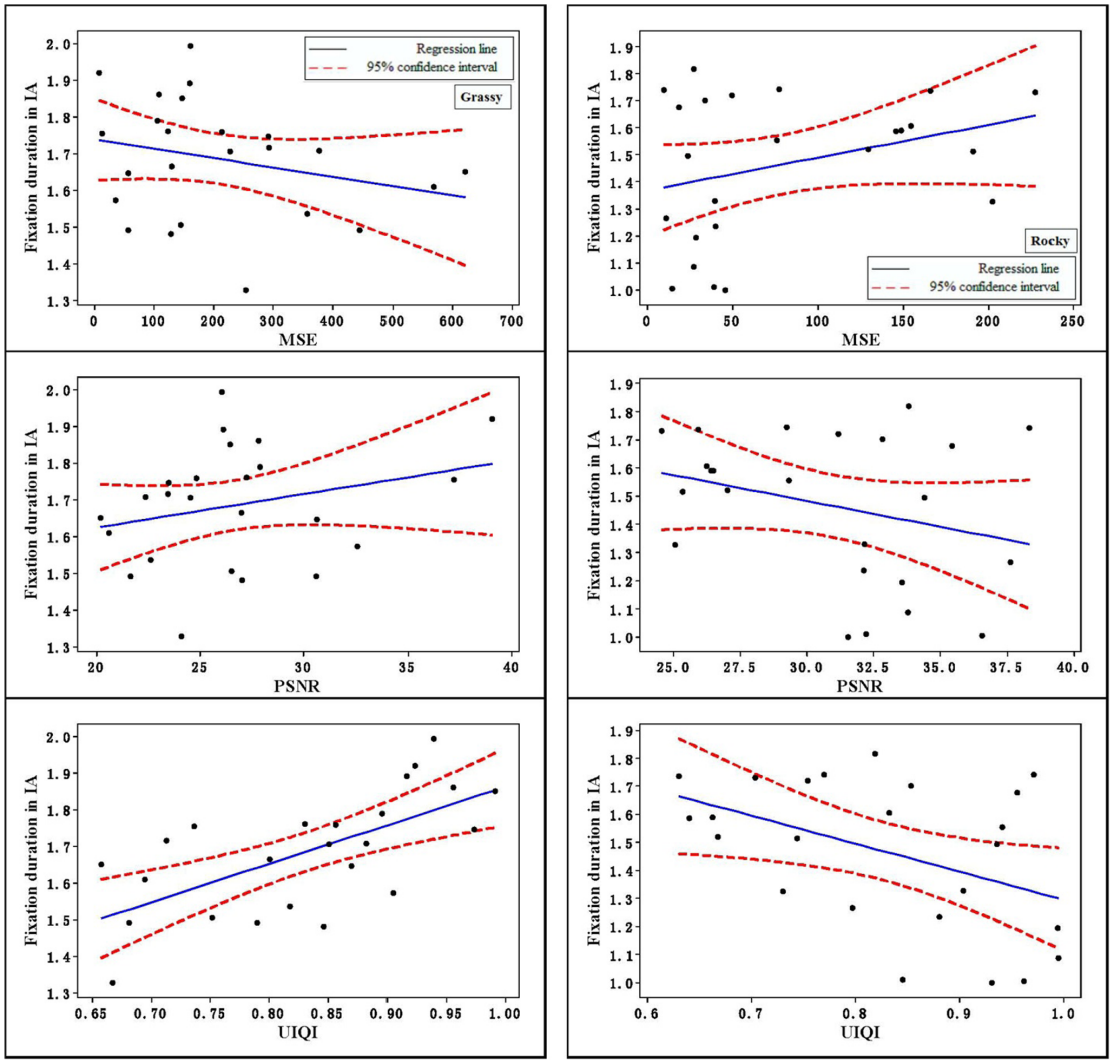
The relationships of MSE, PSNR, and UIQI vs. fixation duration in IA.

Fixation duration in interesting area (IA) was divided by detection time. A higher ratio of fixation duration in the IA indicated that the camouflage target had higher discrimination efficiency. The Pearson correlation coefficient was shown to have significant relationship between the UIQI and fixation duration in the IA on the grassy and rocky backgrounds (as shown in Table 1). Figure 8 shows the mean number of fixations in the interesting area on grassy and rocky background. UIQI had better performance in predicting fixation duration in IA.

## Discussion and Conclusion

In this paper, we report that it is possible to obtain a measure of the similarity between camouflaged targets and backgrounds by using image quality assessment techniques. We used several image quality metrics in common use, the MSE, PSNR and UIQI, to compare targets having different degrees of camouflage with backgrounds. A camouflage search experiment with eye-movement was used in this study to compare the prediction performance of the proposed metrics, and different camouflage patterns on different battlefields were used to analyze visual performance and each of the metrics to understand what kinds of metrics have the greatest effect. Pearson correlation modeling showed that these metrics could be used to predict human performance accuracy to a statistically significant degree, and UIQI outperformed both the MSE and PSNR. The performance also showed that not only UIQI but MSE also had a better prediction for difficulty and first saccade amplitude. Since MSE and UIQI were calculated based on the variance in luminance, it seemed that the difference in luminance had attracted the peripheral vision and guided the saccade to the target, resulting in a better prediction. On the other hand, the first saccade amplitude also correlates with the difficulty rating, implying subjective difficulty was associated with saccade. The fixation duration in IA results showed that MSE and PSNR had poor effects, perhaps due to the lack of structural similarity. That is why the UIQI was a better predictor than the other metrics. From the hit rate and detection time, we found that the MSE and PSNR had poor effects, but we could see that the MSE had better performance in the first saccade. This finding also tells us that if some feature metrics were added to the MSE, it could possibly be a good predictor. From the result, we could find that the eye movement analysis provided good information for use in designing a good algorithm.

In conclusion, in this paper, we report that camouflage similarity is easy to calculate based on UIQI, and it is applicable to many different camouflage patterns. Our measures also provide good results on visual searching support for making the metrics a good predictor of human response. This study also had some limitations. First, there is a ceiling effect in our data. The relationship between those performance measures and our metrics contains a leveling part because many of the camouflage stimuli in the experimental task were quite easy for human observers that they got it right all the time, very quickly, and reported it as being very easy. This may result in a situation that the Pearson correlation coefficients may be significant, but not especially strong. If the experimental task could be designed in a range of more appropriate difficulty, the data would be exhibited without ceiling effect. This can be considered in the future by including different backgrounds and more camouflage stimuli. Despite this ceiling effect, the data still show that in both the rocky and the grass backgrounds the new metric UIQI outperformed the others. Second, the research of this study should be treated with caution when applied to real battle environments, since the experiment was set in a laboratory situation. Potential safety hazards on battlefields, combined with the tremendous psychological and physical workloads of combatants, would no doubt alter how they interact with camouflaged targets and backgrounds. In addition, since eye movement data can be used to describe and analyze each algorithm effect, this data can be used to obtain more information for us in developing or designing camouflage assessment algorithms in the future.

## References

[1] Kilian JC, Hepfinger L (1992) Computer-based evaluation of camouflage. In Proc. SPIE 1687: 358–369.

[2] O’Neill T, Johnsmeyer W, Brusitus J, Taylor D (1977) Psychometric correlates of camouflage target detection. U.S. Military Academy, Office of Institutional Research.

[3] O’Neill T, Johnsmeyer W, Brusitus J, Taylor D (1977) Field evaluation of Dual Texture Gradient Camouflage. U.S. Military Academy, Office of Institutional Research.

[4] Toet A (2000) Executive Summary. Proceedings of the RTO Workshop on Search and Target Acquisition RTO-MP: 45.

[5] Boyce SJ, Pollatsek A (1992) Identification of objects in scenes: The role of scene background in object naming. Journal of Experimental Psychology: Learning, Memory, and Cognition 18: 531–543. 10.1037//0278-7393.18.3.531 1534354

[6] Doll TJ, McWhorter S, Schmieder DE (1993) Target and background characterization based on a simulation of human pattern perception. Proceedings of the SPIE International.

[7] Hogervorst MA, Toet A, Jacobs P (2010) Design and evaluation of (urban) camouflage. SPIE 7662: 1–11.

[8] Muller T, Muller M (2007) Compuer-aided camouflage assesment in real-time. In Proc.SPIE 6543: 701–711.

[9] Li L, Liu J, Fu C, Wu Z (2008) The camouflage evaluation model based on slack-based measure of super-efficiency DEA. In Proceeding of the 2008 Second International Symposium on Intelligent Information Technology Application 2: 181–185.

[10] Williams SB, Pizarro O, How M, Mercer D, Marshall J, et al.. (2009) Surveying noctural cuttlefish camouflage behaviour using an AUV. Robotics and Auomation, ICRA’09, IEEE Inernational Conference 214–219.

[11] Kreis R (2004) Issues of spectral quality in clinical H-magnetic resonance spectroscopy and a gallery of artifacts, NMR in Biomedecine 17361–381. 10.1002/nbm.891 15468083

[12] Avcibas I, Sankur B, Sayood K (2002) Statistical evaluation of image quality measures, Journal of Electronic Imaging. 11: 206–223.

[13] Farrell JE (1999) Image quality evaluation. In Color Imaging: Vision and Technology, eds. L.W. Macdonald and M. R. Luo, Wiley Press 285–313.

[14] Cadik M, Slavik P (2004) Evaluation of two principal approaches to objective image quality assessment. 8th International Conference on Information Visualisation, IEEE Computer Society Press 513–551.

[15] Engeldrum PG (1999) Image quality modeling: where are we? IS&T’s 1999 PICS Conference 251–255.

[16] Wang Z, Bovik AC (2002) A universal image quality index. IEEE Signal Processing Letters. 9: 81–84.

[17] Wang Z, Bovik AC, Sheikh HR, Simoncelli EP (2004) Image quality assessment: from error visibility to structural similarity, IEEE Trans. on Image Processing 13. 10.1109/tip.2003.819861 15376593

[18] Watson AB, Solomon JA (1997) Psychophysica: Mathematica notebooks for psychophysical experiments. Spatial Vision 10: 447–466. 9176955 10.1163/156856897x00384

[19] Piella G, Heijmans H (2003) A new quality metric for image fusion, IEEE Conference on Image Processing, 3, 173–176.

[20] Chang CC, Lee YH, Lin CJ, Liu BS, Shih YC (2012) Visual assessment of camouflaged targets. Perceptual and Motor Skills 114: 1–15. 10.2466/24.PMS.114.2.527-541 22755458

[21] Lin CJ, Chang CC, Lee YH (2013) Evaluating Camouflage Design Using Eye Movement Data. Applied Ergonomics. In press (http://dx.doi.org/10.1016/j.apergo.2013.09.012). 10.1016/j.apergo.2013.09.012 24139724

[22] Alain H, Djemel Z (2010) Image quality metrics: PSNR vs. SSIM. 2010 International Conference on Pattern Recognition 10: 2366–2369.

[23] Doll TJ, Home R (1999) Lessons Learned in Developing and Validating Models of Visual Search and Target Acquisition. NATO/Research and Technology Organization (RTO) Meeting Proceedings 45: 1–8.

[24] Santos LD, Townes DE, Patricio GR, Winterhalter CA, Dugas A, et al.. (2004) Camouflage U.S. Marine corps utility uniform: pattern, fabric, and design. U.S. Patent No. 6,805,957, October 19.

[25] Research and Technology Organisation (RTO) of NATO (2006) Guidelines for camouflage assessment using observers (Publication No. AG-SCI-095). Available: http://ftp.rta.nato.int/public/PubFullText/RTO/AG/RTO-AG-SCI-095/AG-SCI-095-TOC.pdf.

[26] U.S. Army Natick Soldier Research, Development and Engineering Center Natick (2009) Photosimulation camouflage detection test (Publication No.01760–5020). Available: http://zh.scribd.com/doc/19823845/Photosimulation-Camouflage-Detection-Test.

[27] Copeland AC, Trivedi MM (2001) Computational models for search and discrimination. Optical Engineering 40: 1885–1895.

[28] Poole A, Ball LJ (2005) Eye tracking in human-computer interaction and usability research: Current status and future prospects. In C. Ghaoui (Ed.), Encyclopedia of human computer interaction. Hershey, PA: Idea Group 211–219.

[29] Ehmke C, Wilson S (2007) Identifying web usability problems from eye-tracking data. Paper presented at the British Computer Society Conference on Human-Computer Interaction.

[30] Just MA, Carpenter PA (1976) Eye fixations and cognitive processes. Cognitive Psychology 8: 441–480.

[31] Findlay JM (1997) Saccade target selection during visual search. Vision Research 37: 617–631. 9156206 10.1016/s0042-6989(96)00218-0

[32] Goldberg HJ, Wichansky AM (2003) Eye tracking in usability evaluation: A practitioner’s guide. In J. Hyönä, R. Radach, & H. Deubel (Eds.). The mind’s eye: Cognitive and applied aspects of eye movement research. Amsterdam: Elsevier 493–516.

